# Who Should Pay for Global Health, and How Much?

**DOI:** 10.1371/journal.pmed.1001392

**Published:** 2013-02-19

**Authors:** Luis R. Carrasco, Richard Coker, Alex R. Cook

**Affiliations:** 1 Department of Biological Sciences, National University of Singapore, Singapore; 2 Communicable Diseases Policy Research Group, London School of Hygiene and Tropical Medicine, Thailand; 3 Saw Swee Hock School of Public Health, National University of Singapore and National University Health System, Singapore; 4 Department of Statistics and Applied Probability, National University of Singapore, Singapore; 5 Program in Health Services and Systems Research, Duke-NUS Graduate Medical School Singapore, Singapore

## Abstract

Roman Carrasco and colleagues propose a “cap and trade" system for global health involving a cost-effectiveness criterion and a DALY global credit market, mirroring global carbon emission permits trading markets to mitigate climate change.

**Summary Points**

- Mechanisms to establish the expected financial contribution from each country to achieve the health Millennium Development Goals (MDGs) could encourage scaling-up of contributions.
- Mirroring global carbon permit markets to mitigate climate change, we propose a cap-and-trade system consisting of a global cost-effectiveness criterion and a disability-adjusted life year (DALY) global credit market.
- Under this system, high-income and middle-income countries should contribute, respectively, 74% and 26% of the additional US$36–US$45 billion annually needed to attain the health MDGs. The change relative to current contributions would vary, with some countries needing to scale-up substantially their expected annual contributions under the proposed market (e.g., US, US$7–US$10 billion; China, US$2–US$3 billion; Japan, US$2 billion; Germany, US$1.5–US$2 billion), while a few already meet or exceed their required contributions (i.e., Norway, the United Arab Emirates, Luxembourg, and the UK).
- A DALY tradable credit market offers the potential to increase the efficiency of global health investments while promoting international obligations to the pursuit of an agreed global common good.

## Global Health: A Public Good without a Collective-Choice Rule

Global health is a public good and ill-health in one part of the globe has consequences elsewhere: witness recent emerging infectious diseases. It follows that by contributing to global health, donor countries can benefit substantially: directly, in the form of a reduction in communicable disease emergence and transmission [1]–[3], and indirectly, through macroeconomic interactions, trade, travel, migration, reduced threats to food security, environmental degradation, and unsustainable consumption patterns [4]. For instance, reports indicate that incorporating global health into US foreign policy has enhanced American national security and prosperity [5].

Despite the substantial benefits that could be derived from global health and the existence of highly cost-effective global health interventions—e.g., childhood immunisation programmes involving second opportunity measles vaccination or malaria control through high coverage artemisinin combination treatments [6]–[10]—global health continues to be underfunded.

That most low-income countries have achieved insufficient or no progress towards meeting the health Millennium Development Goals (MDGs) by the 2015 target [11] indicates the current level of funding is likely insufficient. An estimated additional US$36 to US$45 billion annually by 2015 is said to be required to meet the health MDGs [11]. This situation is exacerbated by the ongoing global financial crisis that is leading to a slowdown in the growth of bilateral donations [12].

The absence of a mechanism to encourage—or enforce—any expected contributions from each country has, therefore, led to the tragedy of the commons, defined as the depletion of shared resources when users act in a self-interested and independent manner [13], which in this context leads to global health being underfunded because the benefits of contributing to global health are shared with other countries, including those who refrain from donating.

Global health, represented herein as meeting the health MDGs, falls into a category of large-scale global commons such as climate change mitigation. For global commons to be effectively managed requires a unanimous agreement or treaty for a collective-choice rule, such as the Kyoto Protocol (Box 1) [14]. However, for global health, a collective-choice rule to establish the expected contributions from each country is currently lacking.

**Box 1. Cap-and-Trade Measures for Climate Change Mitigation**

**Origins**: Attaining global support for a treaty to tackle climate change was a slow process. Awareness of the threat of climate change and the idea of limiting warming started in the 1970s through a series of scientific and economic reports [27]. Early political developments started in a few countries in the 1980s with reports focusing on the creation of emissions targets. Wide support in preparation for Kyoto was finally obtained in the Second World Climate Conference in Geneva in 1990 [27].

**Design**: The Kyoto Protocol was adopted in the third session of the Conference of the Parties of the United Nations Framework Convention on Climate Change in 1997. It sets legally binding limits on greenhouse gas emissions on signatories. The protocol introduced flexible mechanisms such as Emissions Trading and the Clean Development Mechanism—project-based emissions reductions based for instance in renewable energies in low-income countries. The efficiency generated by having a market of carbon emissions permits stems from the idea that emission abatement costs are much lower in low-income countries (this represents a clear analogy with policies aimed at global disease burden reductions), i.e., it is cheaper for high-income countries to support carbon sequestration projects in low-income countries than to invest in relatively more expensive measures to cut emissions domestically, thereby reducing the economic impact of emissions reduction.

**Outcomes:** The market size of carbon emissions grew from US$11 billion in 2005 to US$140 billion in 2009 where it stalled, influenced by the global financial crisis [28]. The volume of carbon traded in 2008 was 4.8 Gigatonnes (Gt) of CO2e [28], where half of the trade corresponded to actual emissions reductions [29]. Although these outcomes indicate the potential of the cap-and-trade mechanisms, the volume of carbon emissions reductions is still far from the 50 GtCO2e/year needed to stabilize the concentration of CO2e at 550 ppm by 2050 [29]. Challenges include proving the integrity of carbon credits and the excessive allocation of allowances for carbon emissions to some middle-income countries.

## Parallels with Tradable Carbon Permits: Global Health Permits?

**Figure 1:**
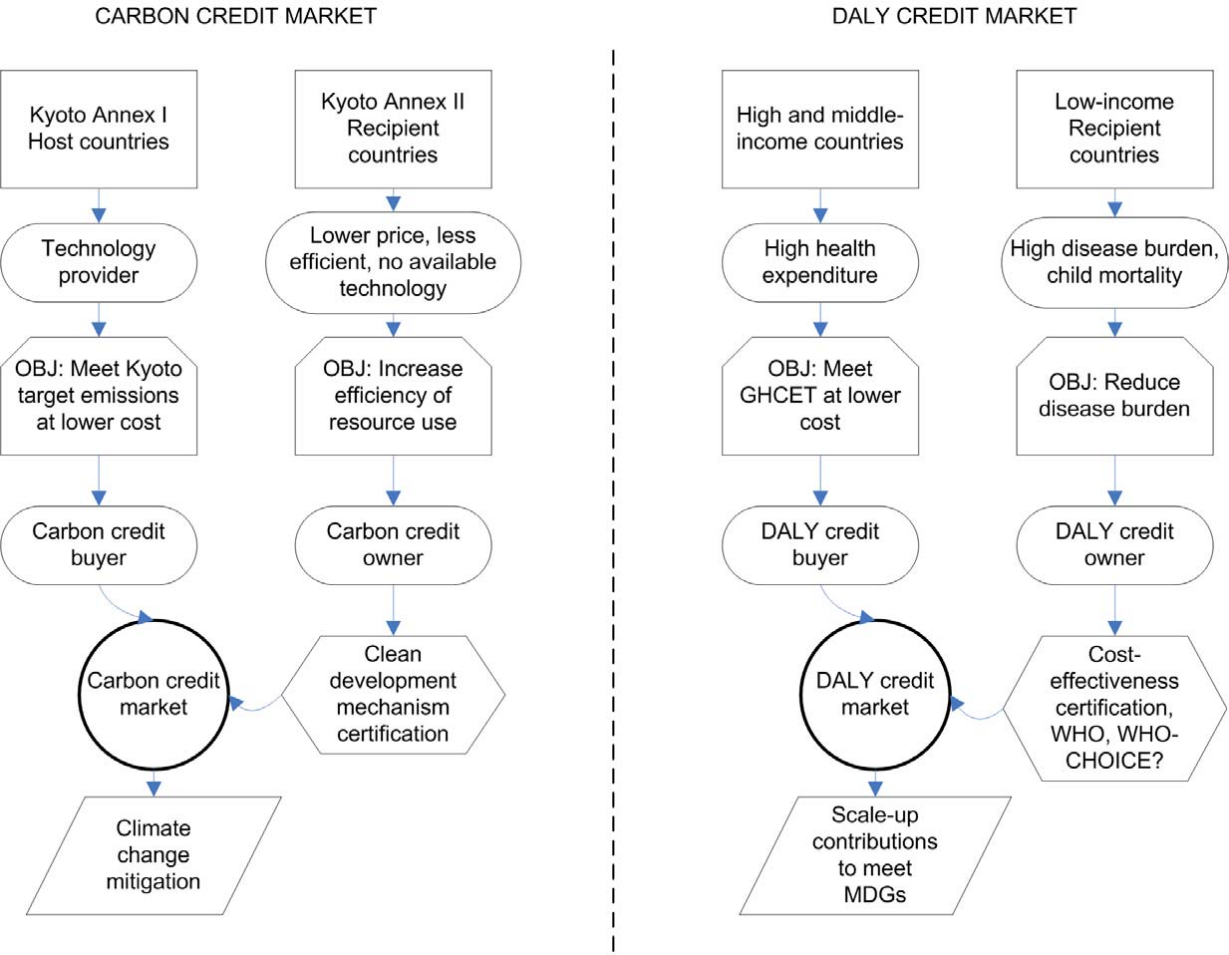
Conceptual comparison of carbon credit markets for the mitigation of climate change and the proposed DALY credit market to meet the health MDGs. OBJ, objective.

Humanity faces new challenges to manage global commons and only one planet to experiment with, so it is important to draw lessons from other successful strategies on global commons management [14]. Market-based systems of tradable carbon emission permits have become one of the bases of the Kyoto agreement on climate change (Box 1). Tradable permits are economically very efficient, making them advantageous compared to command and control measures (Box 1; Figure 1) [15]. A market is completed by a cap-and-trade mechanism: carbon emissions of countries are capped and as a result countries need to buy permits that compensate for their emissions in excess of the cap (Figure 1).

In the case of carbon emission permits, the metric is tonnes of carbon emissions avoided. In our case of global health, a suitable metric is disability-adjusted life years (DALYs) potentially averted [16],[17]. In the Kyoto protocol, the cap rule is based on a global emission target and a consensus of how to apportion responsibility to individual countries, in our case, raising the necessary funds to meet the health MDGs and a rule to share the effort to achieve this reduction. There are many possible ways to set expected contributions from cap-and-trade rules. The method we use here is to link global health aid donations with domestic health investment using the perspective of a hypothetical global social planner aiming to reduce global disease burden: the greater the investment on low cost-effectiveness interventions in high- and middle-income countries, the more inefficient the allocation of resources to reduce global disease burden. The system then encourages compensation for the resulting inefficiency by requiring the support of highly cost-effective projects in low-income countries. Other cap-and-trade frameworks could be adopted and this particular proposal is not necessary for the overall scheme to succeed.

In our proposal, the cap is based on the cost-effectiveness of a health intervention that can be used to identify inefficient levels of health expenditure [18]. A general cost-effectiveness criterion suggested by the Commission on Macroeconomics and Health of the World Health Organization (WHO) [19] recommends that interventions be cost-effective if their cost per avoided DALY in a specified setting is lower than thrice the per capita gross national income (GNI).

To define the cap rule of a tradable DALY credit system, we propose a global health cost-effectiveness threshold (GHCET) under which health interventions are deemed cost-effective, this being three times the GNI threshold classifying countries as low-income. A global DALY tradable permit market would be established, in which high- and middle-income nations who wish to implement an intervention that is cost-effective domestically, but does not meet the GHCET, can purchase averted DALYs from highly cost-effective health interventions in low-income countries (Figure 1 and Box 2 for examples of the system at the project level and Text S1 for details).

**Box 2. Examples of DALY Credit Transactions at the Project Level**

Under the proposed system, the DALY credits that need to be purchased per intervention or project (*N_credits_*) are:
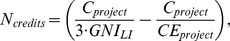
where *C_project_* is the cost of the health intervention, *GNI_LI_* the per capita gross national income threshold by which a country is categorized as low-income, and *CE_project_* the cost-effectiveness of the health intervention. At 2009 dollars, 3·*GNI_LI_* = US$3,015.

Examples of projects that might require offsetting by DALY credits:

1. **Pneumococcal conjugate vaccination in Australia.** Cost-effectiveness is US$100,853 per DALY averted (*CE_project_*), total costs are US$5 million annually [30],[31]. 1,332 DALY credits annually would be required.
2. **Obesity reduction through physician counseling in China and Brazil.** Cost-effectiveness amounts are US$10,300 and US$9,300 per DALY averted, total costs are US$7.4 and US$3.8 million over 20 years [32]. 1,522–751 DALY credits would be required, respectively.
3. **Taxes to reduce tobacco consumption in Western Europe.** Cost-effectiveness is US$51 per DALY averted [28]. Because *CE_project_*<3·*GNI_LI_* no DALY credits are required.
4. **Tuberculosis control in the US.** Cost-effectiveness is US$15 per DALY averted [28]. No DALY credits are required.

Examples of projects that might be offered to the market:

- **Increase in the coverage of the traditional Expanded Program on Immunization in South Asia.** Cost-effectiveness is US$10 per DALY averted [29]. The price of each DALY would be US$10.
- **Second opportunity measles vaccination in sub-Saharan Africa.** Cost-effectiveness is US$5 per DALY averted [29]. The price of each DALY would be US$5.

Examples of transactions to conform to GHCET:

- Project 1 buys credits annually from project A at US$10,654 (increase of 0.21% of the project costs).
- Project 2 buys credits from project B at US$7,600 and US$3,700 in China and Brazil respectively (increase in project costs of 0.1%).

Alternative sources of DALY credits could be multilateral donor agencies (e.g., GAVI, Global Fund, UNITAID) that represent innovative financing mechanisms and improve systems for resource mobilization, pooling, channeling, resource allocation and implementation [33].

Although implementing the trade in averted DALYs would be feasible if cost-effectiveness analyses were universally available for projects in high-, middle-, and low-income countries, at present the cost-effectiveness of many interventions is not known, preventing a global estimation of the volumes that would need to be traded without substantial additional infrastructure. To estimate, in the interim, expected contributions globally, we use a national-level indicator of the difference between the hypothetical DALYs averted domestically and those that could be averted in a low-income setting based on the GNI and health expenditure of each country (see Text S1).

## Expected Contributions

**Figure 2:**
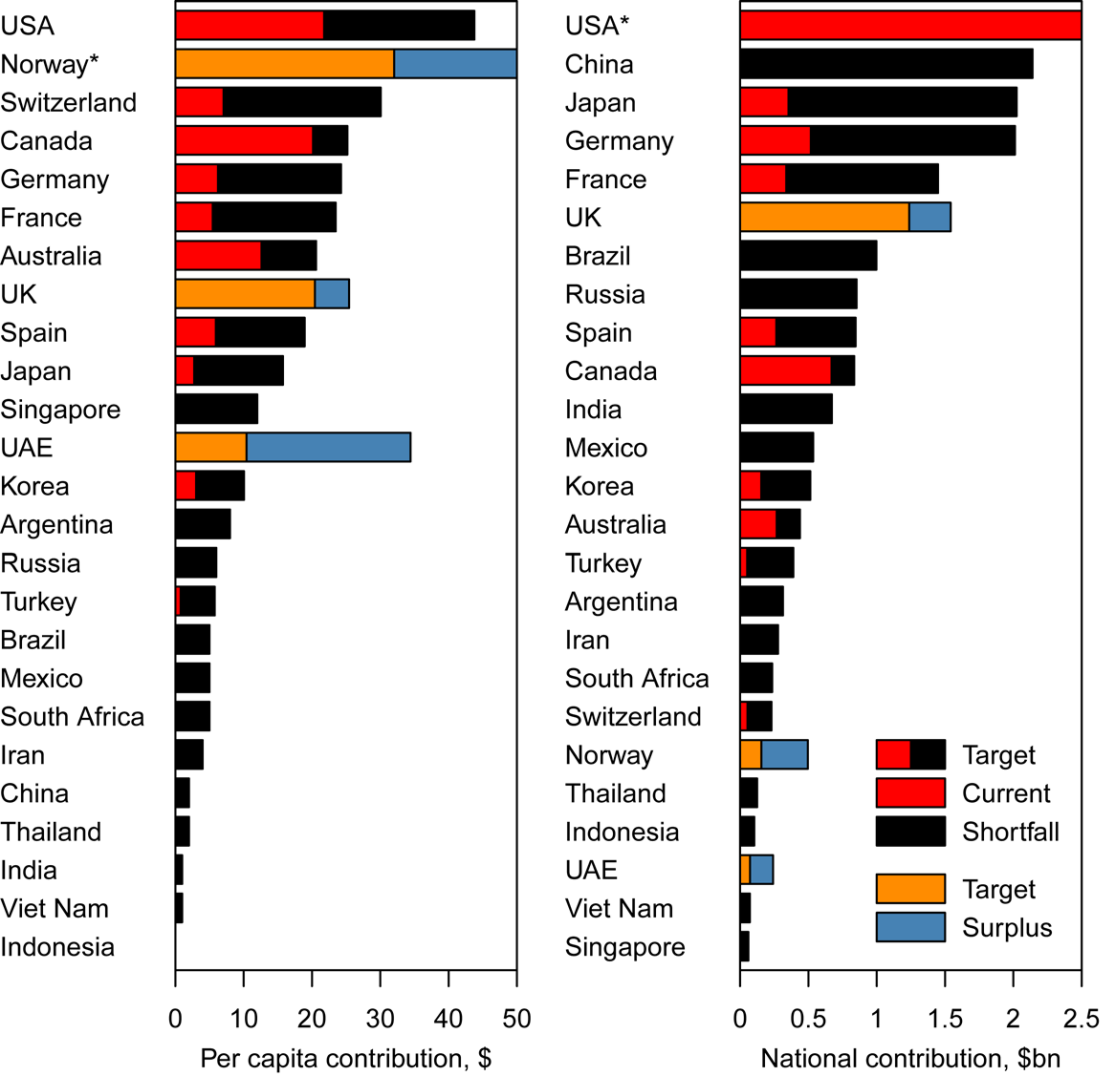
Total and per capita annual expected contributions to meet the health MDGs (“Target"), compared to the current level of donations (“Current") in 25 selected countries. *Norway has an excess of contributions of US$70 per capita, and the US a level of donations of US$6.7 billion and a shortfall of expected contributions of US$6.8 billion; both are off the scale.

Under this proposal, in accordance with their GNI and health expenditure, the greatest defaulting countries per capita to meet the health MDGs were the US (US$22–US$33) and several affluent European countries (e.g., Switzerland, US$23–US$31; Austria, US$21–US$27; and Germany, US$18–US$24 [Figure 2; estimates for all countries can be found in Text S1, Tables S1 and S2]). Only a few countries currently contribute more outlay to global health per capita than would be expected from the DALY credit system (Text S1, Tables S1 and S2): Ireland, the UK, Denmark, the United Arab Emirates, Luxembourg, and Norway.

Under the proposed DALY credit system, to bridge the funding gap between current contributions and the contributions needed to meet the health MDGs, high-income countries would account for 74%–77% of the remaining US$36–US$45 billion in investment required to meet the health MDGs, the rest coming from middle-income countries. 19%–28% of the total increase, or US$6.8–US$10 billion, would come from the US, 5%–6% from Japan, 4%–6% from Germany, 3%–4% from France (Figure 2 and Text S1, Tables S1 and S2), while some of the bigger middle-income countries would also contribute substantially, with 6%–7% from China (i.e., US$2.1–US$2.7 billion), 3% from Brazil, and 2% from India (Figure 2 and Text S1, Tables S1 and S2). Our proposal, therefore, involves a marked change in perspective over who should contribute to meeting the health MDGs, with contributions expected from large emerging economies such as China and Brazil.

Our estimates of necessary increases in health aid are dwarfed if compared with the annual military budgets of many of the countries involved. For instance, the US's global health contributions should increase an equivalent to 1% of its military budget, for Japan and Germany 3%, Brazil 4%, and the increase would be 2% of their military budgets for France, China, and India [20]. We have already noted the security benefits of investing in global health [5].

## Increased Efficiency and Effectiveness in Global Health Allocations

Our proposal is that countries be expected to contribute a number of DALY permits. Differently priced permits would create an incentive for nations to invest permits on the most cost-effective projects—to reduce their financial outlay—enhancing the efficiency of global health allocations (Box 2). This allocation strategy generates a new, more efficient, ranking of allocation priorities very different to current allocations (for an analogous re-ranking process see [21]), which is relevant because the lack of success in achieving the health MDGs is not only caused by insufficiency of funding but also by inefficiency in funding allocation that does not necessarily prioritize the most needy recipients [22],[23].

Scaling-up of contributions and efficiency in their allocation would not, however, mean that global health implementation and efficacy problems such as the temporary nature of financing, lack of delivery coordination [22], fragmentation, or divergence from national policies [23] would be solved. Nonetheless, the system provides for an opportunity to mitigate these problems. For instance, if a centralised DALY market were to be established, donations could be globally coordinated. As a result, transaction costs could be minimized and interventions could match recipient needs better.

## Policy Implementation and Challenges

### Establishing International Support

The system requires countries to relinquish some sovereignty over global health, and the experience of Kyoto suggests the steps involved in achieving this (see Box 1).

First, the system would need to attract the attention of a nucleus of countries, probably those already investing substantial sums on global health. Then, a request by those countries to consider a proposal based on the system would be initiated in a Kyoto-style Conference of the Parties. If an agreement is reached and a legally binding document proposed, countries could opt to sign it. Such agreement would need to provide consensus inter alia over the regulating body, the GHCET (or alternative cap-and-trade rules) and mechanisms to validate DALY credits.

There is undoubtedly a risk that countries could opt to free ride the system by not signing the agreement. However, the proposal would function if a group of altruistic countries willing to contribute proactively towards meeting the health MDGs and countries willing to initiate cooperation hoping that it will be returned [14] would start trading DALY permits, thus exerting peer pressure on other countries. If the system is legally established, this group of countries might grow further with those unwilling to cooperate unless legally assured [14]. Experience from carbon permits suggests success is possible even without full participation: even though the US, Afghanistan, Andorra, and South Sudan are not signatories of the Kyoto protocol, a majority of countries are already participating in fully functioning emissions permit markets. Indeed, cap-and-trade systems for climate change mitigation have shown the potential for the market to grow rapidly (from US$11 to US$140 billion from 2005 to 2011; Box 1). Given the lower volumes required to meet the health MDGs (US$36–US$45 billion increase); scaling-up of global health donations might be a feasible goal.

### Management and Monitoring

The system could be overseen by an international organization where proposals of cost-effective interventions could be submitted for evaluation. Approved projects would be allocated DALY credits, which would be available for purchase by donor countries, NGOs, and philanthropic organizations. The WHO would be a natural choice as overseer and while it would likely be supported by proponents that, in a globalised world, the authority of WHO needs to increase [24], others might dissent. If consensus over the regulating body is not reached, the system could initially be implemented on a regional basis or through voluntary schemes. A decentralized approach in which individual health projects can purchase DALY credits to conform to GHCET (Box 2), on the other hand, would not present as many challenges. Using the current global health architecture, decentralized markets would allow flexibility for global health donors—either at national or project level—to purchase credits from projects of their choice. This approach would, however, require comprehensive cost-effectiveness analyses of projects in different regions to certify their validity as DALYs credits. The WHO-CHOICE project [25] and the Disease Control Priorities Project [26] have covered numerous diseases and regions and would be a solid starting point. Expanding the certification of cost-effectiveness of new projects could, however, impose additional transaction costs.

## Challenges

We have proposed tying global health contributions to national health expenditure via a GHCET. The justification for this is in terms of a hypothetical global planner aiming to prevent the tragedy of the commons in global health by requesting compensation for inefficient domestic allocations. Invoking a global social planner is necessary given a fundamental difference with carbon markets: carbon emissions create direct impacts worldwide but domestic health investment does not. Other cap-and-trade frameworks could be adopted and may receive more support. The GHCET system can be argued against for (i) penalising high expenditure in health and thus creating a disincentive on domestic health investment. This disincentive would likely be small (e.g., a ratio of health expenditure to expected contributions of 1 to 0.0045 occurs for the US); (ii) using a cost-effectiveness criterion that was initially intended only for low-income countries. We favoured its use for all countries because of its simplicity and transparency, despite the relationship between health investment, GNI and health being complex; and (iii) the specific value of the GHCET, which we based on the threshold of low-income countries. Our results would change if that threshold were changed for instance to include middle-income countries (see sensitivity analysis in Text S1). Alternatives to the GHCET would be to base expected DALY contributions on other metrics of ability to pay (e.g., GDP) and need (e.g., poverty or mortality rates) or by attempting to quantify the positive externalities countries would gain and generate from global and domestic health funding and link DALY contributions to that. However, the estimation of those externalities would be challenging. In addition, data on health donations per country are incomplete because countries also contribute to global health indirectly, for instance through tax exemptions for private foundations, and these contribution channels are not readily quantifiable. Prior to the implementation of the system the cap-and-trade rules and formulas determining contributions should be subject of political debate.

The establishment of cooperation would not be immune to global economic crises leading to reductions of global health contributions [12], and indeed the system would be especially vulnerable to economic crises during its implementation period. It is notable, though, that during the current economic crisis only one country (Canada) has withdrawn from its commitments to the Kyoto protocol. A legally binding commitment, and an efficient and transparent market, may be the best protection from the fickleness of global health donations.

## Conclusions

In both theory and practice, we believe experiences from carbon permit markets are encouraging. They efficiently raise resources to help manage global commons, in this case climate change. If implemented, an analogous tradable DALY credits market would incentivise countries to scale-up their global health commitments to meet the health MDGs and, we expect, any post-MDG targets such as the proposed Rio +20 Sustainable Development Goals. If the health MDGs are to be realised, collectively we should be ready to implement the most powerful strategies to manage global commons. A DALY tradable credit market offers the potential to increase the efficiency of global health investments while, at the same time, promoting international obligations to the pursuit of an agreed global common good.

## Supporting Information

Text S1 **Description of the cap-and-trade system.**
(DOC)

## Abbreviations

DALY: disability-adjusted life year
GHCET: global health cost-effectiveness threshold
GNI: gross national income
MDG: Millennium Development Goal
UN: United Nations
WHO: World Health Organization
