# Supplementary material for: Who Should Pay for Global Health, and How Much?
Source: PLoS Med. 2013 Feb 19;10(2):e1001392. doi: 10.1371/journal.pmed.1001392 (PMC3576343; doi:10.1371/journal.pmed.1001392)
Supplement: Text S1 — Description of the cap-and-trade system. (DOC) [file pmed.1001392.s001.doc]

**Supporting Information**

**Description of the cap-and-trade system**

*Cap-and-trade at the project level*

The DALY credits that need to be purchased per intervention or project (*Ncredits*) are estimated as:

$N_{credits}=\left(\frac{C_{project}}{3\cdot GNI_{LI}}-\frac{C_{project}}{CE_{project}}\right)$,

where *Cproject* are the costs of the health intervention; *GNILI* is the per capita gross national income upper threshold by which a country is categorized as low-income; and *CEproject* is the cost-effectiveness of the health intervention.

*Illustration of the system at the project level*

If the DALY credit market were implemented at the project level, the amount of credits to be purchased would be determined by the cost-effectiveness of the project in the high or middle-income country. For instance a health intervention involving $5 million in the US with a cost-effectiveness of $20 000 per DALY averted would be cost-effective by national standards (3∙*GNIi* is ca. $140 000 which is greater than $20 000), however it would not meet the GHCET of $3015 per DALY averted (3∙*GNILI*). The difference between the DALYs averted (250) and potentially averted (1658) with both cost-effectiveness ratios respectively, and the amount of credits to be purchased, would thus be 1408. The cost of these credits could vary widely depending of the project supported in low-income settings. They could be valued for instance at $25/credit for vitamin A, zinc fortification and measles immunization in sub-Saharan Africa . Using these prices for the credits, the increase in costs to the project would range between 0.7 to 3.7% of its total cost, maximizing the efficiency of the allocation of resources. The system would thus penalize more those projects that are not highly cost-effective and would not require the purchase of credits for projects that are below the GHCET. Examples of specific projects are provided in Box 2 of the main manuscript.

*Cap-and-trade at the global level*

Because they do not have the same moral international obligations as national governments, donations from philanthropic organizations and private donations were excluded from the system. To estimate expected contributions per country according to the GHCET, we define:

$$E_{i}=\left(\frac{C_{Hi}}{3\cdot GNI_{LI}}-\frac{C_{Hi}}{3\cdot GNI_{i}}\right)$$

where *Ei* denotes the “force of expected contribution to global health” by country *i*; *CHi* is the annual health expenditure; and *GNIi* is the per capita gross national income of the focus country. *Ei* measures the difference between the hypothetical DALYs that would be averted in a low-income country with cost-effectiveness equal to the threshold for low-income countries using the national health expenditure, compared to the hypothetical DALYs that would be averted in the country using the national cost-effectiveness threshold. We then scale this indicator to estimate the proportion of credits – the expected volume of contributions – to be purchased by each country. Deducting the actual volume of donations per country from their expected contributions then gives the net contribution deficits and excesses per country:

$$F_{i}=\left(\frac{E_{i}}{\sum_{i=1}^{N} E_{i}}\cdot V\right)-D_{Hi}$$

where *Fi* > 0 indicates the annual level of defaulting on global health contributions from the country *i*; *N* are the total number of countries; *V* is the total volume of the necessary increase in global health contributions to meet the health MDGs ($36–45bn ); and *DHi* are the total health commitments of country *i* per year.

*Data*

The data regarding aid donations were collected by the Organisation for Economic Co-operation and Development . We used data on the quantities donated in the categories “total health” and “population policies/programmes and reproductive health” through official development aid by all channels in high and middle-income countries. Total expenditure on health for 2009 was obtained from the WHO . All the data used for the analysis correspond to 2009 and costs are expressed in US $ of 2009.

**Sensitivity analysis**

We conducted univariate sensitivity analysis to estimate the influence of the parameters used in the determination of expected contributions. We chose the United States as an example.

We created uniform distributions ranging from a 10% decrease and a 10% increase of the original values for the parameters which were derived from the literature: *GNILI*, *GNIi*, *CHi­*, *V* and *DHi*. The uniform distributions were sampled 100000 times using Monte Carlo methods and Latin Hypercube sampling. The changes in the mean of the expected contributions were recorded (Figure S1).

Expected contributions were sensitive to the total volume of the necessary increase in global health contributions to meet the health MDGs, followed by the domestic health expenditure, the level of donations, the per capita gross national income upper threshold by which a country is categorized as low-income (*GNILI*) and the GNI of the country.

Figure S1. Sensitivity analysis of expected contributions from the US to DALY market parameters.

**
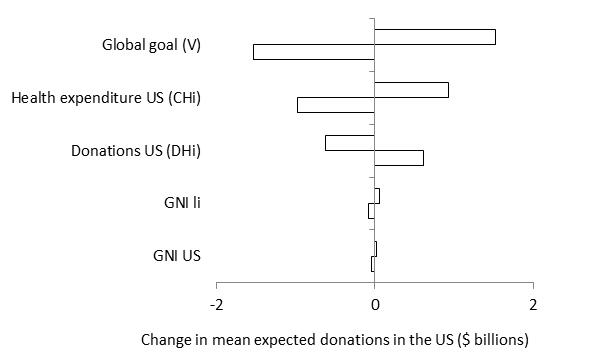
**

**Table S1. Lower bound expected contributions to meet the Millennium Development Goals.** Total expenditure on health on 2009 (CH, $ million ), expected contribution to global health aid (ED, $ million), actual donations in 2009 (DH, $ million), deficit from expected contribution (F, $ million) and deficit from expected contribution per capita (Fp, $) for all countries. All values are expressed in US $ of 2009.

| **Country** | **CH** | **ED** | **DH** | **F** | **Fp** |
| --- | --- | --- | --- | --- | --- |
| Afghanistan | 2110 | 0 | 0 | 0 | 0 |
| Albania | 1858 | 9.98 | 0 | 9.98 | 3.13 |
| Algeria | 19013 | 101.24 | 0 | 101.24 | 2.90 |
| Andorra | 265 | 0 | 0 | 0 | 0 |
| Angola | 5863 | 28.74 | 0 | 28.74 | 1.55 |
| Antigua and Barbuda | 83 | 0.48 | 0 | 0.48 | 5.45 |
| Argentina | 55567 | 313.65 | 0 | 313.65 | 7.83 |
| Armenia | 743 | 3.68 | 0 | 3.68 | 1.19 |
| Australia | 74074 | 438.48 | 268 | 170.24 | 7.77 |
| Austria | 35504 | 210.15 | 34 | 176.58 | 21.10 |
| Azerbaijan | 5086 | 27.47 | 0 | 27.47 | 3.03 |
| Bahamas | 553 | 0 | 0 | 0 | 0 |
| Bahrain | 1821 | 0 | 0 | 0 | 0 |
| Bangladesh | 7057 | 15.08 | 0 | 15.08 | 0.10 |
| Barbados | 397 | 0 | 0 | 0 | 0 |
| Belarus | 7034 | 39.38 | 0 | 39.38 | 4.09 |
| Belgium | 45170 | 267.01 | 209 | 57.58 | 5.40 |
| Belize | 38 | 0 | 0 | 0 | 0 |
| Benin | 559 | 1.14 | 0 | 1.14 | 0.13 |
| Bhutan | 196 | 0.96 | 0 | 0.96 | 1.35 |
| Bolivia | 2082 | 9.66 | 0 | 9.66 | 0.99 |
| Bosnia and Herzegovina | 3500 | 18.84 | 0 | 18.84 | 5.00 |
| Botswana | 2657 | 14.89 | 0 | 14.89 | 7.51 |
| Brazil | 182232 | 998.06 | 0 | 998.06 | 5.16 |
| Brunei Darussalam | 582 | 0 | 0 | 0 | 0 |
| Bulgaria | 7437 | 41.78 | 0 | 41.78 | 5.54 |
| Burkina Faso | 1407 | 1.21 | 0 | 1.21 | 0.08 |
| Burundi | 400 | 0 | 0 | 0 | 0 |
| Cambodia | 1663 | 4.53 | 0 | 4.53 | 0.32 |
| Cameroon | 2339 | 7.69 | 0 | 7.69 | 0.40 |
| Canada | 141302 | 835.69 | 669 | 166.26 | 4.94 |
| Cape Verde | 86 | 0.37 | 0 | 0.37 | 0.76 |
| Central African Republic | 138 | 0 | 0 | 0 | 0 |
| Chad | 1028 | 0.83 | 0 | 0.83 | 0.08 |
| Chile | 19872 | 111.74 | 0 | 111.74 | 6.59 |
| China | 412487 | 2141.43 | 0 | 2141.43 | 1.60 |
| Colombia | 25977 | 139.44 | 0 | 139.44 | 3.05 |
| Comoros | 31 | 0.03 | 0 | 0.03 | 0.04 |
| Congo | 497 | 0 | 0 | 0 | 0 |
| Cook Islands | 8 | 0 | 0 | 0 | 0 |
| Costa Rica | 5348 | 29.52 | 0 | 29.52 | 6.43 |
| Croatia | 6850 | 39.46 | 0 | 39.46 | 8.95 |
| Cuba | 5665 | 0.00 | 0 | 0 | 0 |
| Cyprus | 1990 | 11.69 | 0 | 11.69 | 10.72 |
| Czech Republic | 20086 | 116.96 | 9 | 108.08 | 10.35 |
| Côte d'Ivoire | 1664 | 3.92 | 0 | 3.92 | 0.20 |
| Democratic People's Republic of Korea | 0 | 0 | 0 | 0 | 0 |
| Democratic Republic of the Congo | 449 | 1.83 | 0 | 1.83 | 0.03 |
| Denmark | 22751 | 134.70 | 202 | -67.67 | -12.25 |
| Djibouti | 141 | 0.51 | 0 | 0.51 | 0.59 |
| Dominica | 43 | 0.23 | 0 | 0.23 | 3.35 |
| Dominican Republic | 4849 | 25.82 | 0 | 25.82 | 2.64 |
| Ecuador | 7174 | 38.19 | 0 | 38.19 | 2.68 |
| Egypt | 22480 | 112.46 | 0 | 112.46 | 1.41 |
| El Salvador | 2631 | 13.49 | 0 | 13.49 | 2.19 |
| Equatorial Guinea | 942 | 5.43 | 0 | 5.43 | 7.97 |
| Eritrea | 66 | 0 | 0 | 0 | 0 |
| Estonia | 1842 | 10.61 | 0 | 10.61 | 7.91 |
| Ethiopia | 3248 | 0 | 0 | 0 | 0 |
| Fiji | 141 | 0.67 | 0 | 0.67 | 0.78 |
| Finland | 17932 | 105.88 | 50 | 56.27 | 10.53 |
| France | 245658 | 1448.93 | 338 | 1111.03 | 17.79 |
| Gabon | 758 | 4.24 | 0 | 4.24 | 2.87 |
| Gambia | 141 | 0.21 | 0 | 0.21 | 0.12 |
| Georgia | 2182 | 10.43 | 0 | 10.43 | 2.38 |
| Germany | 340252 | 2011.68 | 518 | 1493.42 | 18.12 |
| Ghana | 2907 | 6.06 | 0 | 6.06 | 0.25 |
| Greece | 34943 | 204.97 | 21 | 183.52 | 16.20 |
| Grenada | 65 | 0.34 | 0 | 0.34 | 3.28 |
| Guatemala | 4729 | 22.42 | 0 | 22.42 | 1.60 |
| Guinea | 595 | 0 | 0 | 0 | 0 |
| Guinea-Bissau | 52 | 0.02 | 0 | 0.02 | 0.01 |
| Guyana | 194 | 0.82 | 0 | 0.82 | 1.09 |
| Haiti | 700 | 0 | 0 | 0 | 0 |
| Honduras | 1713 | 7.59 | 0 | 7.59 | 1.02 |
| Hungary | 14413 | 82.99 | 0 | 82.99 | 8.30 |
| Iceland | 977 | 5.75 | 0 | 5.75 | 18.24 |
| India | 159422 | 672.08 | 0 | 672.08 | 0.56 |
| Indonesia | 23504 | 104.26 | 0 | 104.26 | 0.44 |
| Iran (Islamic Republic of) | 50099 | 277.83 | 0 | 277.83 | 3.80 |
| Iraq | 5131 | 21.77 | 0 | 21.77 | 0.71 |
| Ireland | 17671 | 104.14 | 124 | -19.60 | -4.44 |
| Israel | 15096 | 88.34 | 0 | 88.34 | 12.17 |
| Italy | 182373 | 1073.52 | 119 | 954.74 | 15.85 |
| Jamaica | 1046 | 5.47 | 0 | 5.47 | 2.00 |
| Japan | 343335 | 2024.10 | 354 | 1669.73 | 13.19 |
| Jordan | 3007 | 15.07 | 0 | 15.07 | 2.50 |
| Kazakhstan | 8776 | 48.15 | 0 | 48.15 | 3.04 |
| Kenya | 2683 | 5.87 | 0 | 5.87 | 0.15 |
| Kiribati | 29 | 0.12 | 0 | 0.12 | 1.25 |
| Kuwait | 3964 | 0 | 0 | 0 | 0 |
| Kyrgyzstan | 801 | 2.65 | 0 | 2.65 | 0.50 |
| Lao People's Democratic Republic | 526 | 1.74 | 0 | 1.74 | 0.28 |
| Latvia | 2252 | 12.91 | 0 | 12.91 | 5.71 |
| Lebanon | 4424 | 24.87 | 0 | 24.87 | 5.93 |
| Lesotho | 286 | 0.77 | 0 | 0.77 | 0.36 |
| Liberia | 203 | 0 | 0 | 0 | 0 |
| Libyan Arab Jamahiriya | 4440 | 25.33 | 0 | 25.33 | 4.05 |
| Lithuania | 3665 | 20.98 | 0 | 20.98 | 6.28 |
| Luxembourg | 3248 | 19.41 | 47 | -27.46 | -55.17 |
| Madagascar | 825 | 0 | 0 | 0 | 0 |
| Malawi | 722 | 0 | 0 | 0 | 0 |
| Malaysia | 18922 | 106.58 | 0 | 106.58 | 3.81 |
| Maldives | 128 | 0.63 | 0 | 0.63 | 2.02 |
| Mali | 984 | 0.93 | 0 | 0.93 | 0.06 |
| Malta | 1770 | 10.29 | 0 | 10.29 | 24.79 |
| Marshall Islands | 16 | 0 | 0 | 0 | 0 |
| Mauritania | 159 | 0.47 | 0 | 0.47 | 0.14 |
| Mauritius | 943 | 5.30 | 0 | 5.30 | 4.10 |
| Mexico | 94780 | 534.79 | 0 | 534.79 | 4.77 |
| Micronesia (Federated States of) | 43 | 0 | 0 | 0 | 0 |
| Monaco | 266 | 0 | 0 | 0 | 0 |
| Mongolia | 453 | 1.92 | 0 | 1.92 | 0.71 |
| Morocco | 7940 | 37.24 | 0 | 37.24 | 1.18 |
| Mozambique | 1143 | 0 | 0 | 0 | 0 |
| Myanmar | 1095 | 0 | 0 | 0 | 0 |
| Namibia | 861 | 4.40 | 0 | 4.40 | 1.96 |
| Nauru | 2 | 0 | 0 | 0 | 0 |
| Nepal | 2031 | 1.83 | 0 | 1.83 | 0.06 |
| Netherlands | 72679 | 430.58 | 250 | 180.54 | 10.90 |
| New Zealand | 11528 | 67.54 | 25 | 42.34 | 9.79 |
| Nicaragua | 1450 | 5.33 | 0 | 5.33 | 0.93 |
| Niger | 599 | 0 | 0 | 0 | 0 |
| Nigeria | 21010 | 65.70 | 0 | 65.70 | 0.43 |
| Niue | 4 | 0 | 0 | 0 | 0 |
| Norway | 26079 | 155.64 | 496 | -340.57 | -70.45 |
| Oman | 2134 | 0 | 0 | 0 | 0 |
| Pakistan | 10741 | 40.80 | 0 | 40.80 | 0.24 |
| Palau | 21 | 0 | 0 | 0 | 0 |
| Panama | 3742 | 20.87 | 0 | 20.87 | 6.03 |
| Papua New Guinea | 476 | 1.61 | 0 | 1.61 | 0.24 |
| Paraguay | 1934 | 9.09 | 0 | 9.09 | 1.43 |
| Peru | 11506 | 61.28 | 0 | 61.28 | 2.13 |
| Philippines | 12472 | 54.28 | 0 | 54.28 | 0.59 |
| Poland | 51981 | 298.58 | 0 | 298.58 | 7.81 |
| Portugal | 28817 | 167.84 | 9 | 158.53 | 14.87 |
| Qatar | 3339 | 0 | 0 | 0 | 0 |
| Republic of Korea | 87726 | 513.53 | 155 | 358.35 | 7.47 |
| Republic of Moldova | 1229 | 4.97 | 0 | 4.97 | 1.38 |
| Romania | 16648 | 94.20 | 0 | 94.20 | 4.37 |
| Russian Federation | 148501 | 853.11 | 0 | 853.11 | 5.96 |
| Rwanda | 1052 | 0.71 | 0 | 0.71 | 0.07 |
| Saint Kitts and Nevis | 43 | 0 | 0 | 0 | 0 |
| Saint Lucia | 134 | 0 | 0 | 0 | 0 |
| Saint Vincent and the Grenadines | 57 | 0.30 | 0 | 0.30 | 2.79 |
| Samoa | 57 | 0.26 | 0 | 0.26 | 1.45 |
| San Marino | 117 | 0 | 0 | 0 | 0 |
| Sao Tome and Principe | 23 | 0.06 | 0 | 0.06 | 0.39 |
| Saudi Arabia | 30830 | 179.55 | 0 | 179.55 | 6.70 |
| Senegal | 1235 | 3.34 | 0 | 3.34 | 0.28 |
| Serbia and Montenegro | 8236 | 45.76 | 0 | 45.76 | 4.64 |
| Seychelles | 71 | 0.41 | 0 | 0.41 | 4.72 |
| Sierra Leone | 608 | 0 | 0 | 0 | 0 |
| Singapore | 10317 | 61.44 | 0 | 61.44 | 12.42 |
| Slovakia | 10348 | 60.04 | 0 | 60.04 | 11.01 |
| Slovenia | 5012 | 29.30 | 0 | 29.30 | 14.48 |
| Solomon Islands | 77 | 0.21 | 0 | 0.21 | 0.41 |
| Somalia | 0 | 0 | 0 | 0 | 0 |
| South Africa | 42886 | 234.60 | 0 | 234.60 | 4.72 |
| Spain | 143760 | 845.90 | 265 | 581.01 | 12.73 |
| Sri Lanka | 3989 | 19.08 | 0 | 19.08 | 0.92 |
| Sudan | 6839 | 20.58 | 0 | 20.58 | 0.48 |
| Suriname | 285 | 0 | 0 | 0 | 0 |
| Swaziland | 365 | 1.75 | 0 | 1.75 | 1.50 |
| Sweden | 34358 | 203.32 | 141 | 62.54 | 6.72 |
| Switzerland | 38655 | 229.93 | 54 | 176.31 | 23.13 |
| Syrian Arab Republic | 2767 | 13.16 | 0 | 13.16 | 0.66 |
| Tajikistan | 712 | 2.10 | 0 | 2.10 | 0.31 |
| Thailand | 23704 | 125.12 | 0 | 125.12 | 1.82 |
| The former Yugoslav Republic of Macedonia | 1541 | 8.50 | 0 | 8.50 | 4.13 |
| Timor-Leste | 132 | 0 | 0 | 0 | 0 |
| Togo | 336 | 0 | 0 | 0 | 0 |
| Tonga | 24 | 0.12 | 0 | 0.12 | 1.12 |
| Trinidad and Tobago | 2316 | 13.51 | 0 | 13.51 | 10.11 |
| Tunisia | 5431 | 28.76 | 0 | 28.76 | 2.78 |
| Turkey | 69332 | 390.03 | 52 | 337.74 | 4.70 |
| Turkmenistan | 712 | 3.70 | 0 | 3.70 | 0.74 |
| Tuvalu | 3 | 0 | 0 | 0 | 0 |
| Uganda | 3722 | 3.52 | 0 | 3.52 | 0.11 |
| Ukraine | 20343 | 103.54 | 0 | 103.54 | 2.26 |
| United Arab Emirates | 12185 | 72.59 | 241 | -168.12 | -24.23 |
| United Kingdom | 209556 | 1238.00 | 1541 | -302.56 | -4.91 |
| United Republic of Tanzania | 2960 | 4.70 | 0 | 4.70 | 0.11 |
| United States of America | 2279959 | 13552.64 | 6741 | 6811.65 | 22.14 |
| Uruguay | 3287 | 18.42 | 0 | 18.42 | 5.49 |
| Uzbekistan | 4123 | 16.41 | 0 | 16.41 | 0.60 |
| Vanuatu | 41 | 0.19 | 0 | 0.19 | 0.81 |
| Venezuela (Bolivarian Republic of) | 7843 | 43.75 | 0 | 43.75 | 1.53 |
| Viet Nam | 18510 | 71.98 | 0 | 71.98 | 0.83 |
| Yemen | 3313 | 11.45 | 0 | 11.45 | 0.49 |
| Zambia | 865 | 1.13 | 0 | 1.13 | 0.09 |
| Zimbabwe | 699 | 0 | 0 | 0 | 0 |

**Table S2. Upper bound expected contributions to meet the Millennium Development Goals.** Total expenditure on health on 2009 (CH, $ million), expected contribution to global health aid (ED, $ million), actual donations in 2009 (DH, $ million), deficit from expected contribution (F, $ million) and deficit from expected contribution per capita (Fp, $) for all countries. All values are expressed in US $ of 2009.

| **Country** | **CH** | **ED** | **DH** | **F** | **Fp** |
| --- | --- | --- | --- | --- | --- |
| Afghanistan | 2110 | 0 | 0 | 0 | 0 |
| Albania | 1858 | 12.48 | 0 | 12.48 | 3.91 |
| Algeria | 19013 | 126.55 | 0 | 126.55 | 3.62 |
| Andorra | 265 | 0 | 0 | 0 | 0 |
| Angola | 5863 | 35.92 | 0 | 35.92 | 1.94 |
| Antigua and Barbuda | 83 | 0.60 | 0 | 0.60 | 6.81 |
| Argentina | 55567 | 392.06 | 0 | 392.06 | 9.79 |
| Armenia | 743 | 4.60 | 0 | 4.60 | 1.49 |
| Australia | 74074 | 548.10 | 268 | 279.86 | 12.78 |
| Austria | 35504 | 262.69 | 34 | 229.11 | 27.37 |
| Azerbaijan | 5086 | 34.34 | 0 | 34.34 | 3.79 |
| Bahamas | 553 | 0 | 0 | 0 | 0 |
| Bahrain | 1821 | 0 | 0 | 0 | 0 |
| Bangladesh | 7057 | 18.85 | 0 | 18.85 | 0.13 |
| Barbados | 397 | 0 | 0 | 0 | 0 |
| Belarus | 7034 | 49.23 | 0 | 49.23 | 5.11 |
| Belgium | 45170 | 333.77 | 209 | 124.33 | 11.66 |
| Belize | 38 | 0 | 0 | 0 | 0 |
| Benin | 559 | 1.42 | 0 | 1.42 | 0.17 |
| Bhutan | 196 | 1.20 | 0 | 1.20 | 1.69 |
| Bolivia | 2082 | 12.08 | 0 | 12.08 | 1.24 |
| Bosnia and Herzegovina | 3500 | 23.55 | 0 | 23.55 | 6.25 |
| Botswana | 2657 | 18.61 | 0 | 18.61 | 9.39 |
| Brazil | 182232 | 1247.57 | 0 | 1247.57 | 6.46 |
| Brunei Darussalam | 582 | 0 | 0 | 0 | 0 |
| Bulgaria | 7437 | 52.22 | 0 | 52.22 | 6.92 |
| Burkina Faso | 1407 | 1.51 | 0 | 1.51 | 0.09 |
| Burundi | 400 | 0 | 0 | 0 | 0 |
| Cambodia | 1663 | 5.66 | 0 | 5.66 | 0.40 |
| Cameroon | 2339 | 9.62 | 0 | 9.62 | 0.50 |
| Canada | 141302 | 1044.62 | 669 | 375.18 | 11.14 |
| Cape Verde | 86 | 0.46 | 0 | 0.46 | 0.95 |
| Central African Republic | 138 | 0 | 0 | 0 | 0 |
| Chad | 1028 | 1.04 | 0 | 1.04 | 0.10 |
| Chile | 19872 | 139.67 | 0 | 139.67 | 8.24 |
| China | 412487 | 2676.79 | 0 | 2676.79 | 2.01 |
| Colombia | 25977 | 174.30 | 0 | 174.30 | 3.82 |
| Comoros | 31 | 0.04 | 0 | 0.04 | 0.05 |
| Congo | 497 | 0 | 0 | 0 | 0 |
| Cook Islands | 8 | 0 | 0 | 0 | 0 |
| Costa Rica | 5348 | 36.90 | 0 | 36.90 | 8.04 |
| Croatia | 6850 | 49.32 | 0 | 49.32 | 11.18 |
| Cuba | 5665 | 0 | 0 | 0 | 0 |
| Cyprus | 1990 | 14.62 | 0 | 14.62 | 13.41 |
| Czech Republic | 20086 | 146.20 | 9 | 137.32 | 13.15 |
| Côte d'Ivoire | 1664 | 4.90 | 0 | 4.90 | 0.25 |
| Democratic People's Republic of Korea | 0 | 0 | 0 | 0 | 0 |
| Democratic Republic of the Congo | 449 | 2.29 | 0 | 2.29 | 0.04 |
| Denmark | 22751 | 168.38 | 202 | -33.99 | -6.15 |
| Djibouti | 141 | 0.64 | 0 | 0.64 | 0.73 |
| Dominica | 43 | 0.28 | 0 | 0.28 | 4.19 |
| Dominican Republic | 4849 | 32.28 | 0 | 32.28 | 3.29 |
| Ecuador | 7174 | 47.74 | 0 | 47.74 | 3.35 |
| Egypt | 22480 | 140.57 | 0 | 140.57 | 1.76 |
| El Salvador | 2631 | 16.86 | 0 | 16.86 | 2.74 |
| Equatorial Guinea | 942 | 6.78 | 0 | 6.78 | 9.96 |
| Eritrea | 66 | 0 | 0 | 0 | 0 |
| Estonia | 1842 | 13.26 | 0 | 13.26 | 9.88 |
| Ethiopia | 3248 | 0 | 0 | 0 | 0 |
| Fiji | 141 | 0.83 | 0 | 0.83 | 0.98 |
| Finland | 17932 | 132.36 | 50 | 82.74 | 15.49 |
| France | 245658 | 1811.16 | 338 | 1473.26 | 23.59 |
| Gabon | 758 | 5.29 | 0 | 5.29 | 3.58 |
| Gambia | 141 | 0.26 | 0 | 0.26 | 0.16 |
| Georgia | 2182 | 13.04 | 0 | 13.04 | 2.98 |
| Germany | 340252 | 2514.60 | 518 | 1996.34 | 24.23 |
| Ghana | 2907 | 7.58 | 0 | 7.58 | 0.32 |
| Greece | 34943 | 256.22 | 21 | 234.77 | 20.73 |
| Grenada | 65 | 0.43 | 0 | 0.43 | 4.10 |
| Guatemala | 4729 | 28.03 | 0 | 28.03 | 2.00 |
| Guinea | 595 | 0 | 0 | 0 | 0 |
| Guinea-Bissau | 52 | 0.02 | 0 | 0.02 | 0.01 |
| Guyana | 194 | 1.02 | 0 | 1.02 | 1.36 |
| Haiti | 700 | 0 | 0 | 0 | 0 |
| Honduras | 1713 | 9.49 | 0 | 9.49 | 1.27 |
| Hungary | 14413 | 103.74 | 0 | 103.74 | 10.37 |
| Iceland | 977 | 7.19 | 0 | 7.19 | 22.79 |
| India | 159422 | 840.10 | 0 | 840.10 | 0.70 |
| Indonesia | 23504 | 130.33 | 0 | 130.33 | 0.55 |
| Iran (Islamic Republic of) | 50099 | 347.28 | 0 | 347.28 | 4.75 |
| Iraq | 5131 | 27.22 | 0 | 27.22 | 0.89 |
| Ireland | 17671 | 130.17 | 124 | 6.44 | 1.46 |
| Israel | 15096 | 110.42 | 0 | 110.42 | 15.21 |
| Italy | 182373 | 1341.90 | 119 | 1223.12 | 20.30 |
| Jamaica | 1046 | 6.84 | 0 | 6.84 | 2.51 |
| Japan | 343335 | 2530.13 | 354 | 2175.76 | 17.19 |
| Jordan | 3007 | 18.84 | 0 | 18.84 | 3.13 |
| Kazakhstan | 8776 | 60.18 | 0 | 60.18 | 3.80 |
| Kenya | 2683 | 7.34 | 0 | 7.34 | 0.19 |
| Kiribati | 29 | 0.15 | 0 | 0.15 | 1.57 |
| Kuwait | 3964 | 0 | 0 | 0 | 0 |
| Kyrgyzstan | 801 | 3.31 | 0 | 3.31 | 0.63 |
| Lao People's Democratic Republic | 526 | 2.17 | 0 | 2.17 | 0.35 |
| Latvia | 2252 | 16.14 | 0 | 16.14 | 7.14 |
| Lebanon | 4424 | 31.09 | 0 | 31.09 | 7.41 |
| Lesotho | 286 | 0.96 | 0 | 0.96 | 0.45 |
| Liberia | 203 | 0 | 0 | 0 | 0 |
| Libyan Arab Jamahiriya | 4440 | 31.67 | 0 | 31.67 | 5.06 |
| Lithuania | 3665 | 26.23 | 0 | 26.23 | 7.85 |
| Luxembourg | 3248 | 24.26 | 47 | -22.60 | -45.42 |
| Madagascar | 825 | 0 | 0 | 0 | 0 |
| Malawi | 722 | 0 | 0 | 0 | 0 |
| Malaysia | 18922 | 133.22 | 0 | 133.22 | 4.77 |
| Maldives | 128 | 0.79 | 0 | 0.79 | 2.53 |
| Mali | 984 | 1.16 | 0 | 1.16 | 0.08 |
| Malta | 1770 | 12.87 | 0 | 12.87 | 30.99 |
| Marshall Islands | 16 | 0 | 0 | 0 | 0 |
| Mauritania | 159 | 0.58 | 0 | 0.58 | 0.17 |
| Mauritius | 943 | 6.62 | 0 | 6.62 | 5.13 |
| Mexico | 94780 | 668.48 | 0 | 668.48 | 5.97 |
| Micronesia (Federated States of) | 43 | 0 | 0 | 0 | 0 |
| Monaco | 266 | 0 | 0 | 0 | 0 |
| Mongolia | 453 | 2.40 | 0 | 2.40 | 0.89 |
| Morocco | 7940 | 46.55 | 0 | 46.55 | 1.47 |
| Mozambique | 1143 | 0 | 0 | 0 | 0 |
| Myanmar | 1095 | 0 | 0 | 0 | 0 |
| Namibia | 861 | 5.51 | 0 | 5.51 | 2.46 |
| Nauru | 2 | 0 | 0 | 0 | 0 |
| Nepal | 2031 | 2.29 | 0 | 2.29 | 0.08 |
| Netherlands | 72679 | 538.22 | 250 | 288.18 | 17.40 |
| New Zealand | 11528 | 84.42 | 25 | 59.22 | 13.70 |
| Nicaragua | 1450 | 6.66 | 0 | 6.66 | 1.17 |
| Niger | 599 | 0 | 0 | 0 | 0 |
| Nigeria | 21010 | 82.13 | 0 | 82.13 | 0.53 |
| Niue | 4 | 0 | 0 | 0 | 0 |
| Norway | 26079 | 194.55 | 496 | -301.66 | -62.40 |
| Oman | 2134 | 0 | 0 | 0 | 0 |
| Pakistan | 10741 | 51.00 | 0 | 51.00 | 0.30 |
| Palau | 21 | 0 | 0 | 0 | 0 |
| Panama | 3742 | 26.09 | 0 | 26.09 | 7.54 |
| Papua New Guinea | 476 | 2.01 | 0 | 2.01 | 0.30 |
| Paraguay | 1934 | 11.36 | 0 | 11.36 | 1.79 |
| Peru | 11506 | 76.60 | 0 | 76.60 | 2.66 |
| Philippines | 12472 | 67.85 | 0 | 67.85 | 0.74 |
| Poland | 51981 | 373.23 | 0 | 373.23 | 9.76 |
| Portugal | 28817 | 209.80 | 9 | 200.49 | 18.81 |
| Qatar | 3339 | 0 | 0 | 0 | 0 |
| Republic of Korea | 87726 | 641.92 | 155 | 486.74 | 10.15 |
| Republic of Moldova | 1229 | 6.22 | 0 | 6.22 | 1.73 |
| Romania | 16648 | 117.74 | 0 | 117.74 | 5.47 |
| Russian Federation | 148501 | 1066.39 | 0 | 1066.39 | 7.45 |
| Rwanda | 1052 | 0.88 | 0 | 0.88 | 0.09 |
| Saint Kitts and Nevis | 43 | 0 | 0 | 0 | 0 |
| Saint Lucia | 134 | 0 | 0 | 0 | 0 |
| Saint Vincent and the Grenadines | 57 | 0.38 | 0 | 0.38 | 3.49 |
| Samoa | 57 | 0.33 | 0 | 0.33 | 1.81 |
| San Marino | 117 | 0 | 0 | 0 | 0 |
| Sao Tome and Principe | 23 | 0.08 | 0 | 0.08 | 0.49 |
| Saudi Arabia | 30830 | 224.44 | 0 | 224.44 | 8.37 |
| Senegal | 1235 | 4.17 | 0 | 4.17 | 0.34 |
| Serbia and Montenegro | 8236 | 57.20 | 0 | 57.20 | 5.81 |
| Seychelles | 71 | 0.51 | 0 | 0.51 | 5.90 |
| Sierra Leone | 608 | 0 | 0 | 0 | 0 |
| Singapore | 10317 | 76.80 | 0 | 76.80 | 15.53 |
| Slovakia | 10348 | 75.05 | 0 | 75.05 | 13.76 |
| Slovenia | 5012 | 36.63 | 0 | 36.63 | 18.10 |
| Solomon Islands | 77 | 0.27 | 0 | 0.27 | 0.51 |
| Somalia | 0 | 0 | 0 | 0 | 0 |
| South Africa | 42886 | 293.25 | 0 | 293.25 | 5.89 |
| Spain | 143760 | 1057.37 | 265 | 792.48 | 17.36 |
| Sri Lanka | 3989 | 23.85 | 0 | 23.85 | 1.15 |
| Sudan | 6839 | 25.72 | 0 | 25.72 | 0.61 |
| Suriname | 285 | 0 | 0 | 0 | 0 |
| Swaziland | 365 | 2.19 | 0 | 2.19 | 1.87 |
| Sweden | 34358 | 254.14 | 141 | 113.37 | 12.18 |
| Switzerland | 38655 | 287.42 | 54 | 233.79 | 30.68 |
| Syrian Arab Republic | 2767 | 16.45 | 0 | 16.45 | 0.82 |
| Tajikistan | 712 | 2.62 | 0 | 2.62 | 0.39 |
| Thailand | 23704 | 156.40 | 0 | 156.40 | 2.28 |
| The former Yugoslav Republic of Macedonia | 1541 | 10.62 | 0 | 10.62 | 5.16 |
| Timor-Leste | 132 | 0 | 0 | 0 | 0 |
| Togo | 336 | 0 | 0 | 0 | 0 |
| Tonga | 24 | 0.14 | 0 | 0.14 | 1.40 |
| Trinidad and Tobago | 2316 | 16.89 | 0 | 16.89 | 12.64 |
| Tunisia | 5431 | 35.95 | 0 | 35.95 | 3.47 |
| Turkey | 69332 | 487.54 | 52 | 435.25 | 6.06 |
| Turkmenistan | 712 | 4.63 | 0 | 4.63 | 0.93 |
| Tuvalu | 3 | 0 | 0 | 0 | 0 |
| Uganda | 3722 | 4.40 | 0 | 4.40 | 0.14 |
| Ukraine | 20343 | 129.42 | 0 | 129.42 | 2.83 |
| United Arab Emirates | 12185 | 90.73 | 241 | -149.98 | -21.61 |
| United Kingdom | 209556 | 1547.51 | 1541 | 6.94 | 0.11 |
| United Republic of Tanzania | 2960 | 5.87 | 0 | 5.87 | 0.13 |
| United States of America | 2279959 | 16940.80 | 6741 | 10199.80 | 33.15 |
| Uruguay | 3287 | 23.03 | 0 | 23.03 | 6.86 |
| Uzbekistan | 4123 | 20.51 | 0 | 20.51 | 0.76 |
| Vanuatu | 41 | 0.24 | 0 | 0.24 | 1.02 |
| Venezuela (Bolivarian Republic of) | 7843 | 54.69 | 0 | 54.69 | 1.92 |
| Viet Nam | 18510 | 89.97 | 0 | 89.97 | 1.04 |
| Yemen | 3313 | 14.31 | 0 | 14.31 | 0.61 |
| Zambia | 865 | 1.41 | 0 | 1.41 | 0.11 |
| Zimbabwe | 699 | 0 | 0 | 0 | 0 |

**References**
